# Dynamin-related protein 1 is required for normal mitochondrial bioenergetic and synaptic function in CA1 hippocampal neurons

**DOI:** 10.1038/cddis.2015.94

**Published:** 2015-04-16

**Authors:** L Y Shields, H Kim, L Zhu, D Haddad, A Berthet, D Pathak, M Lam, R Ponnusamy, L G Diaz-Ramirez, T M Gill, H Sesaki, L Mucke, K Nakamura

**Affiliations:** 1 Gladstone Institute of Neurological Disease, San Francisco, CA, USA; 2 Graduate Programs in Neuroscience and Biomedical Sciences, University of California San Francisco, San Francisco, CA, USA; 3 Department of Neurology, University of California, San Francisco, San Francisco, CA, USA; 4 Tetrad Graduate Program, University of California, San Francisco, San Francisco, CA, USA; 5 Gladstone Institute of Cardiovascular Disease, San Francisco, CA, USA; 6 Department of Cell Biology, Johns Hopkins, Baltimore, MD, USA

## Abstract

Disrupting particular mitochondrial fission and fusion proteins leads to the death of specific neuronal populations; however, the normal functions of mitochondrial fission in neurons are poorly understood, especially *in vivo*, which limits the understanding of mitochondrial changes in disease. Altered activity of the central mitochondrial fission protein dynamin-related protein 1 (Drp1) may contribute to the pathophysiology of several neurologic diseases. To study Drp1 in a neuronal population affected by Alzheimer's disease (AD), stroke, and seizure disorders, we postnatally deleted Drp1 from CA1 and other forebrain neurons in mice (CamKII-Cre, Drp1^lox/lox^ (Drp1cKO)). Although most CA1 neurons survived for more than 1 year, their synaptic transmission was impaired, and Drp1cKO mice had impaired memory. In Drp1cKO cell bodies, we observed marked mitochondrial swelling but no change in the number of mitochondria in individual synaptic terminals. Using ATP FRET sensors, we found that cultured neurons lacking Drp1 (Drp1KO) could not maintain normal levels of mitochondrial-derived ATP when energy consumption was increased by neural activity. These deficits occurred specifically at the nerve terminal, but not the cell body, and were sufficient to impair synaptic vesicle cycling. Although Drp1KO increased the distance between axonal mitochondria, mitochondrial-derived ATP still decreased similarly in Drp1KO boutons with and without mitochondria. This indicates that mitochondrial-derived ATP is rapidly dispersed in Drp1KO axons, and that the deficits in axonal bioenergetics and function are not caused by regional energy gradients. Instead, loss of Drp1 compromises the intrinsic bioenergetic function of axonal mitochondria, thus revealing a mechanism by which disrupting mitochondrial dynamics can cause dysfunction of axons.

Mitochondrial dynamics – the balance between mitochondrial fission and fusion – regulates mitochondrial quality control by segregating poorly functioning mitochondria for degradation while mixing the contents of healthy mitochondria.^1, 2^ In neurons, fission uniquely facilitates movement of mitochondria down narrow distal axons.^3, 4^ Disruptions of this movement, and of other neuron-specific functions, may explain why systemic mutations in mitochondrial fusion and fission proteins specifically cause death of neurons. However, the roles and requirements of these proteins also differ between neuronal types.^1^ For example, mutations in the fusion protein optic atrophy 1 cause degeneration of retinal ganglion neurons,^5^ and mutations in the fusion protein mitofusin-2 or the fission protein ganglioside-induced differentiation-associated protein 1 cause peripheral neuropathy (Charcot-Marie-Tooth types 2A and 4A^6, 7^).

There are several potential reasons why specific neurons have unique requirements for fission–fusion proteins. First, the functions of these proteins may be more critical in vulnerable neuronal populations. Recently, we showed that most midbrain DA neurons are uniquely vulnerable to loss of the central mitochondrial fission protein dynamin-related protein 1 (Drp1),^4^ a GTPase recruited to fission sites on the outer mitochondrial membrane.^1^ Loss of Drp1 depletes axonal mitochondria, which is followed by axonal degeneration and neuronal death. However, a subpopulation of midbrain DA neurons survive, despite losing their axonal mitochondria, suggesting that they have lower needs for energy or other mitochondrial functions in their axons.^4^ Do unique requirements for mitochondrial dynamics underlie differential neuronal vulnerability? Do resistant neurons compensate with other fission or fusion mechanisms? Do the functions of fission differ between neurons? Notably, Drp1 may also have mitochondria-independent functions in synaptic vesicle release.^8^ Addressing these issues could help elucidate the physiological functions of mitochondrial dynamics in the nervous system and reveal how shifts in the fission–fusion balance contribute to selective neuronal death in neurodegenerative diseases, including Huntington's disease, Parkinson's disease and Alzheimer's disease (AD),^1, 4^ and in other neurologic disorders, including stroke and epilepsy.^9, 10, 11^

To understand mitochondrial dynamics, it would be useful to know why mitochondrial fission is needed in the nervous system in the first place, and how loss of fission affects mitochondrial functions in specific cell types. Notably, Drp1 knockout did not change respiration or ATP levels in resuspended mouse embryonic fibroblasts (MEFs),^12, 13^ indicating that mitochondrial fission is not required for respiration in these cells. However, neuronal respiration may be more sensitive to Drp1 loss. Indeed, Drp1 loss markedly decreased the number of mitochondria in axons and the cell body in midbrain DA neurons *in vivo*,^4^ and reduced staining of complex I and IV activity in cerebellar neurons *in vivo*.^14^ However, it is unclear whether these changes translate into decreased ATP levels in neurons and, if so, whether this decrease compromises neuronal function. Furthermore, Drp1 loss caused cell death in cerebellar and most midbrain DA neurons,^4, 14^ which challenges our ability to dissociate the specific effects of Drp1 loss on mitochondrial function from other non-specific changes that accompany cell death.

To learn how disrupting mitochondrial fission contributes to selective neurodegeneration, we studied the function of Drp1 in CA1 hippocampal neurons and its role in mitochondrial bioenergetics. Surprisingly, despite losing Drp1, most CA1 neurons survived for more than 1 year *in vivo*, although their function was compromised, leading to deficits in synaptic transmission and memory. To begin to understand how loss of Drp1 causes neuronal dysfunction, we examined the role of Drp1 in mitochondrial bioenergetics. We found that Drp1 is required to maintain normal mitochondrial-derived ATP levels specifically in axons (but not the cell body), and that the loss of this function is unrelated to the distribution of mitochondria within axons.

## Results

To selectively delete Drp1 from CA1 hippocampal neurons, we bred floxed Drp1 mice^13^ with CamKIIalpha (CamKCre) mice, which express Cre recombinase from P19 in a subset of hippocampal neurons, including nearly all CA1 neurons, and in scattered cortical and other neurons throughout the forebrain.^15, 16^ Drp1cKO (Drp1^lox/lox^; CamKII-Cre) mice were the progeny of Drp1^lox/lox^ and Drp1^wt/lox^; CamKII-Cre. Drp1WT included control mice (Drp1^wt/lox^ and Drp1^lox/lox^) lacking the Cre transgene. Drp1cKO mice were born in roughly normal Mendelian proportions (control 46.6%, Drp1 heterozygotes 29.8%, Drp1cKO 23.6%, *n*=191), and no differences in survival were noted. Drp1cKO and control mice had similar body weights through 10 months of age (Supplementary Figure S1a).

To confirm Cre expression in CA1 neurons, we crossed Drp1cKO mice with floxed tdTomato reporter mice.^17^ In 1-month-old tdTomato-CamKCre control (tdTomato^lox/wt^; CamKII-Cre) and Drp1cKO-tdTomato-CamKCre (Drp1^lox/lox^; tdTomato^lox/wt^; CamKII-Cre) mice, ≥98% of CA1 neurons (immunostained for NeuN) showed tdTomato fluorescence, indicating that they expressed Cre (Supplementary Figure S1b). Conversely, all TdTomato+ cells expressed NeuN, indicating that Cre was only expressed in neurons (Supplementary Figure S1c). We found that Cre was expressed in ~50% of cells in the dentate gyrus, though at much lower levels than in CA1 (Supplementary Figure S1d and e). There was very little expression of Cre in CA3 neurons, but it was also expressed in scattered neurons throughout the cortex (not shown). CA1 neurons in Drp1cKO mice had markedly decreased Drp1 expression at 1 year of age (Supplementary Figure S1f and g).

### Drp1 loss decreases CA1 volume but has little effect on the survival of CA1 neurons

**Figure 1:**
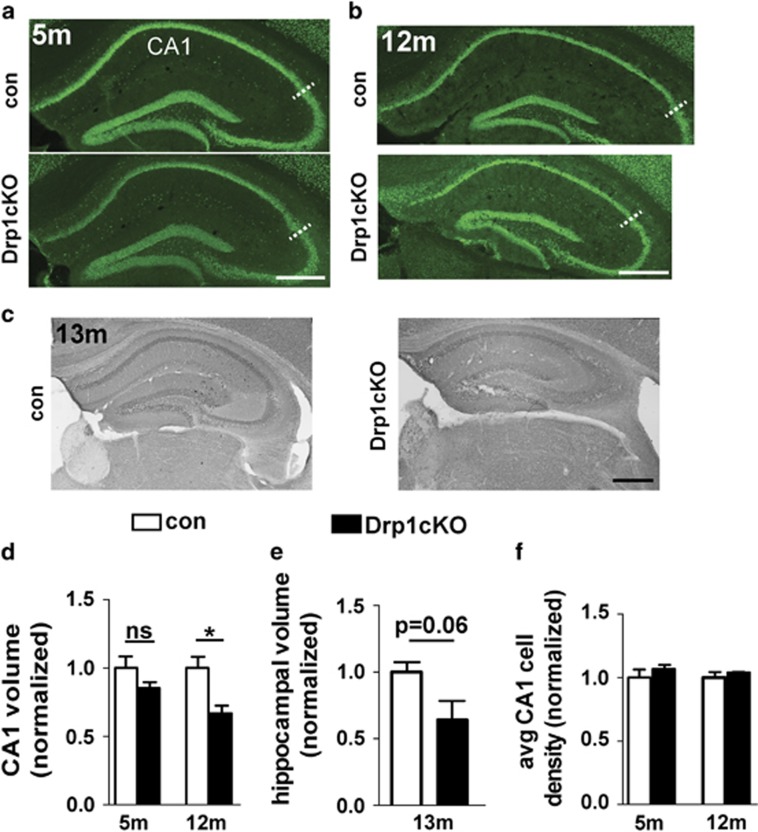
Drp1cKO mice develop atrophy of CA1. (**a** and **b**) NeuN staining of (**a**) 5- and (**b**) 12-month-old Drp1WT (control) and Drp1cKO brain sections, with CA1 defined by calbindin staining (not shown). The lateral margin of CA1 is demarcated by hashed white lines. Scale bars are 400 *μ*m. (**c**) Sections from 13-month-old tdTomato-Drp1cKO (Drp1^lox/lox^; tdTomato^lox/wt^; CamKII-Cre) and tdTomato-CamKCre control (Drp1^wt/wt^; tdTomato^lox/wt^; CamKII-Cre) stained with peroxidase for dsRed to visualize tdTomato+ cells. Scale bar is 400 *μ*m. (**d**) CA1 volume of Drp1cKO mice was similar to controls at 5 months, but significantly decreased by 12 months. Data are means±S.E.M., ns=not significant, *P<0.05 *versus* respective control group by two-way ANOVA and Sidak *post hoc* test, *n*=3 mice/group (with 10–18 slices of hippocampus examined per mouse). (**e**) 13-month-old tdTomato-Drp1cKO mice showed a strong trend toward decreased total hippocampal volume *versus* controls. Data are means±S.E.M.; *P*=0.06 by unpaired two-tailed *t*-test, *n*=3–4 mice/group (with 12–26 slices of hippocampus examined per mouse). (**f**) Drp1cKO showed no change in average CA1 cell density at 5 or 12 months compared with controls. Data are means±S.E.M.; ns by two-way ANOVA, *n*=3–4 mice/group (four slices examined per mouse)

Deleting Drp1 promotes loss of most midbrain DA neurons within 1–2 months,^4^ and most cerebellar Purkinje neurons within 6 months.^14^ To determine whether Drp1cKO similarly compromises survival of CA1 neurons, we examined the effects of Drp1cKO on CA1 volume (Figures 1a, b and d). Surprisingly, at 5 months, CA1 volume did not significantly decrease, and by 1 year, CA1 volume was still ~70% of controls (Figure 1d). There was a similar trend toward decreased volume of the entire hippocampus at 13 months (*P*=0.06) (Figures 1c and e), suggestive of hippocampal atrophy in Drp1cKO mice. The density of NeuN-positive cells within CA1 was not significantly changed even at 1 year (Figure 1f). Considering the decrease in CA1 volume at 1 year, loss of Drp1 may have caused synaptodendritic rarefaction,^18, 19^ in addition to some neuronal death. Nonetheless, most CA1 neurons in Drp1cKO mice survive beyond 1 year, indicating that CA1 hippocampal neurons are more resistant to Drp1 loss than cerebellar Purkinje neurons and most midbrain DA neurons.^4, 14^

### Drp1 is required for normal spatial memory and synaptic function

**Figure 2:**
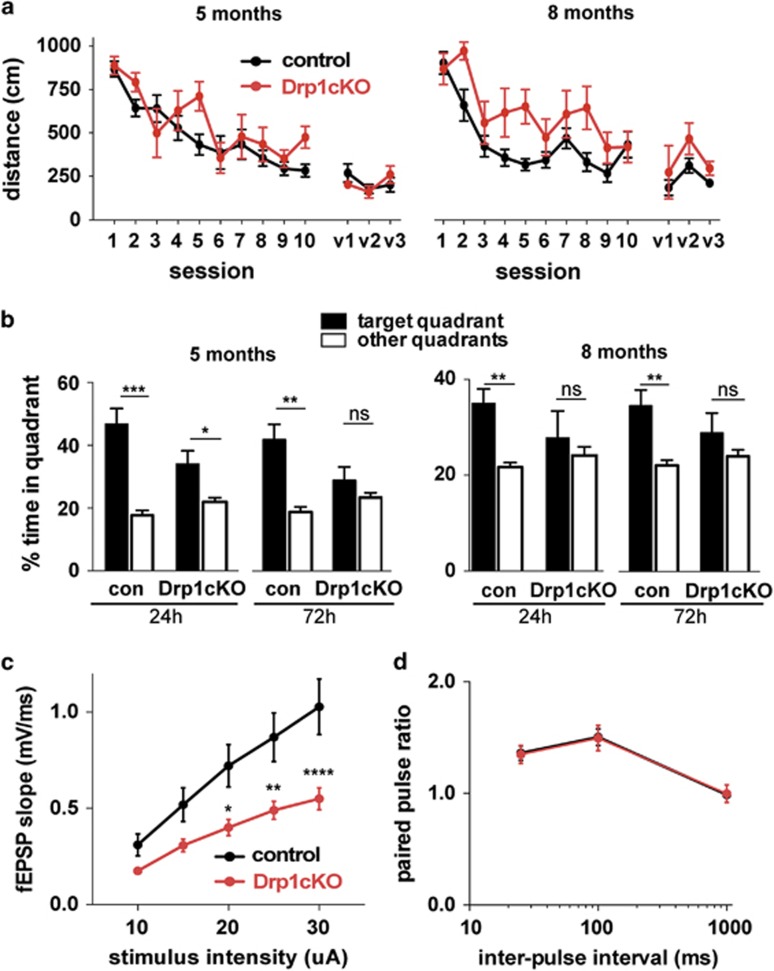
Drp1 loss produces deficits in memory and synaptic transmission. (**a**) Learning was evaluated by Morris water maze (MWM) hidden-platform training results (sessions 1–10). Drp1cKO mice did not show significant learning deficits at 5 or 8 months (analyzed with a linear mixed effects model and Holm *post hoc* test). Visible platform training results (days v1–v3) did not differ between Drp1WT (control) and Drp1cKO. (**b**) Memory was evaluated during MWM probe trials at 24 and 72 h, based on the percent time spent in the target quadrant compared with the average of the other three quadrants. Drp1cKO mice showed memory deficits at both 5 and 8 months. Data are means±S.E.M.; ns=not significant, *P<0.05, **P<0.01, ***P<0.001 by paired one-tailed *t*-test, *n*=7–16 mice/group. (**c** and **d**) The impact of Drp1cKO on the electrophysiologic function of CA1 neurons was assessed in acute hippocampal slices by stimulating the Schaffer collaterals, and performing extracellular field recordings in CA1. At 7–9 months, Drp1cKO mice showed impaired synaptic transmission in CA3-CA1 (input/output slopes), but no change in paired-pulse facilitation compared with Drp1WT (control). Data are means±S.E.M.; *P<0.05, **P<0.01, ****P<0.0001 by two-way ANOVA with repeated-measures and Sidak *post hoc* test, *n*=13–17 slices/group

To determine whether Drp1 is required for normal hippocampal function, we examined how Drp1cKO affects spatial learning and memory in the Morris water maze. Drp1cKO mice showed normal learning at 5 and 8 months, based on an analysis of learning curves with a linear mixed effects model^20^ (Figure 2a and Supplementary Figure S2). However, in probe trials carried out 24 and 72 h after the last training trial, these mice did not favor the target quadrant as well as controls (Figure 2b). Drp1cKO mice performed normally in cued platform trials, had normal swim speeds, and showed no abnormalities in open-field behavior (Figure 2a and Supplementary Figure S3a and b), indicating that their water maze deficits were not caused by deficits in vision or motor function.

Next, we examined the function of CA1 neurons in acute hippocampal slices with extracellular field recordings in CA1 after stimulating the Schaffer collaterals. Drp1cKO mice had markedly lower field excitatory post-synaptic potentials (EPSPs) than controls (Figure 2c). To determine whether these effects result from pre- or post-synaptic changes, we examined paired-pulse ratios, which were unchanged in Drp1cKO slices (Figure 2d). These results suggest that pre-synaptic strength was normal in Drp1cKO mice, consistent with the lack of Cre expression in most CA3 neurons (Supplementary Figure S1d and e). Therefore, deficits in synaptic transmission likely resulted from post-synaptic changes in CA1 pyramidal cells.

### Drp1 loss changes morphology, but not mass or synaptic localization, of mitochondria in CA1 neurons

**Figure 3:**
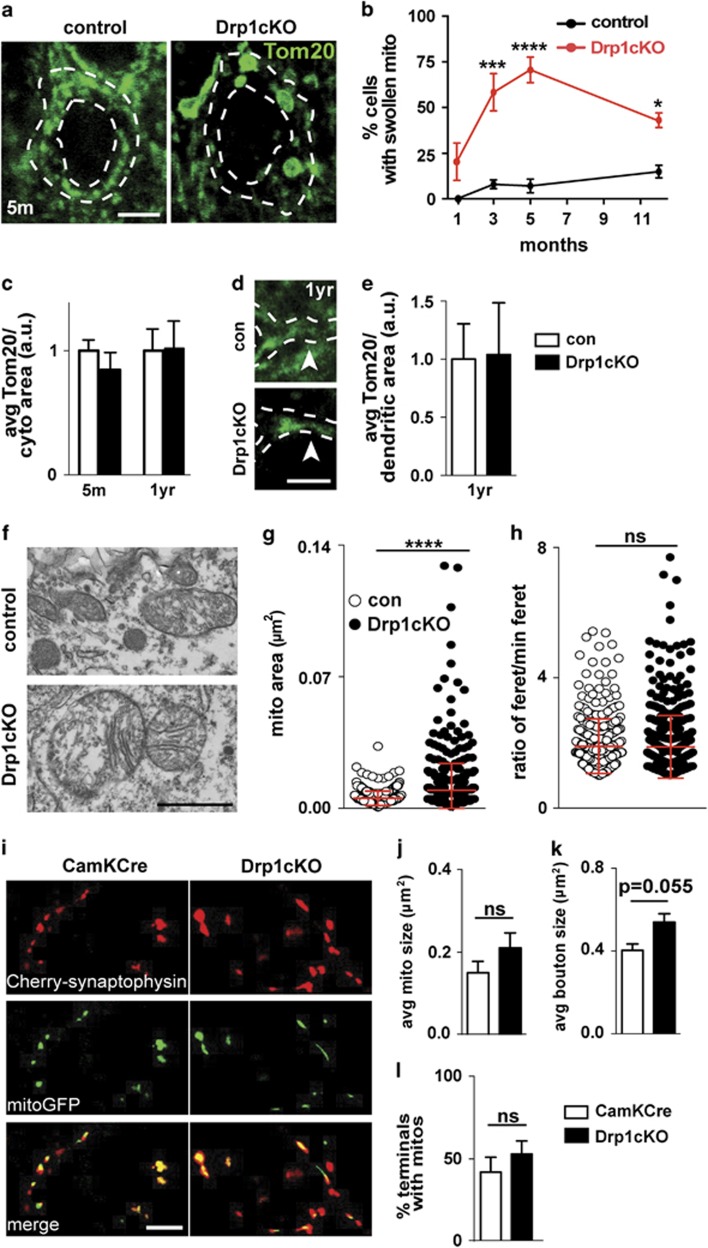
Drp1 loss alters mitochondrial morphology but fails to deplete mitochondria from CA1 synapses. (**a** and **d**) Staining for the mitochondrial marker Tom20 in the CA1 of brain slices from 5-month-old (**a**) and 1-year-old (**d**) Drp1cKO and Drp1WT (control) mice. Outlines of cell bodies and dendrites (indicated by the stippled outlines and arrowheads) and the nucleus (inner stippled circles) were defined by MAP2 immunostaining (not shown). (**b**) CA1 cells showed an increase in the number of swollen mitochondria between 1 and 3 months, though the numbers decreased somewhat by 12 months. Data are means±S.E.M.; *P<0.05, ***P<0.001, ****P<0.0001 *versus* the control group by two-way ANOVA, *n*=3 mice/group (with 18–76 cells/mouse). (**c**) However, CA1 cells did not show any change in mean Tom20 fluorescence over the cytoplasm (cell body, excluding the nucleus) at 5 months or 1 year (**e**), nor in the dendrites at 1 year. Data are means±S.E.M.; not significant by two-way ANOVA (cell body) and unpaired two-tailed *t*-test (dendrites), *n*=3 mice/group (with 3–5 slices/mouse). (**f** and **g**) Ultrastructural analysis of CA1 neurons at the cell body revealed larger mitochondria in Drp1cKO mice than Drp1WT (control). Dots on the scatter graph show the areas of individual mitochondria. (**h**) Mitochondria in Drp1cKO and controls were equally round, as measured by ratio of feret diameter over minimum feret diameter. Red bars show mean±S.D.; ns=not significant, ****P<0.0001 *versus* the control group by unpaired two-tailed *t*-test, *n*=67–223 mitochondria/group. (**i**) AAVs expressing mitochondria-targeted GFP (mitoGFP; green, to visualize mitochondria) and mCherry-synaptophysin (red, to visualize synaptic boutons) in DIO constructs^59^ that express only in Cre-expressing neurons^4^ were co-injected into the CA1 of Drp1cKO (Drp1^lox/lox^; CamKII-Cre) and CamKCre (CamKII-Cre) control mice, and examined at 7 months. (**j**) The size of axonal mitochondria was unchanged by Drp1cKO (**k**), but bouton size showed a trend toward increased size (*P*=0.055). (**l**) The percentage of synaptic boutons containing mitochondria was unchanged. Data are means±S.E.M.; ns=not significant *versus* control group by unpaired two-tailed *t*-test, *n*=3–4 mice/group (total of 86–1279 mitochondria). Scale bars are 5 *μ*m (**a**, **d** and **i**) and 1 *μ*m (**f**)

To understand how Drp1 loss compromises neuronal function, we first examined its effect on mitochondrial morphology and distribution in CA1 neurons. At the cell body, many Drp1cKO neurons developed characteristic swollen mitochondria between 1 and 3 months of age, identified by Tom20 immunofluorescence (Figures 3a and b), similar to those observed after Drp1 deletion in other neuronal types.^4, 13^ This suggests that mitochondrial fission is lost between 1 and 3 months. Surprisingly, the percentage of neurons with swollen mitochondria decreased somewhat between 5 and 12 months (Figure 3b), despite having low levels of Drp1 (Supplementary Figure S1f and g). However, unlike the effects of Drp1 loss in DA neurons,^4^ mitochondrial content (mitochondrial intensity/cytoplasmic area) was unchanged in CA1 neurons, even in 1-year-old animals (Figure 3c). Mitochondrial content was also unchanged in proximal dendrites (Figures 3d and e), and the morphology of dendritic mitochondria was grossly normal despite swollen mitochondria at the cell body. Drp1cKO mitochondria were larger, but their length/width (Feret) ratio was unchanged (Figures 3f–h). Although the cristae structure was intact in most Drp1cKO mitochondria, some larger mitochondria had disrupted cristae (Figure 3f).

Next, we examined the mitochondria in CA1 axons, particularly those that project to the entorhinal cortex.^21^ We visualized the synapses and mitochondria (Figure 3i) by co-injecting Cre-dependent reporter viruses expressing mCherry-synaptophysin and mitoGFP^4^ into CA1 of 6-month-old Drp1cKO (Drp1^lox/lox^; CamKII-Cre) and CamKCre (CamKII-Cre) control mice. Animals were killed at ~7 months, and we confirmed that all injections hit CA1, but not areas outside of the hippocampus (not shown). Mitochondria in Drp1cKO axons were not larger than controls (Figure 3j). There was a strong trend for increased size of boutons (Figure 3k). Interestingly, unlike midbrain DA axons,^4^ the percentage of boutons containing mitochondria was unchanged (Figure 3l), suggesting that mitochondria in CA1 axons target the synapse independent of Drp1.

### Drp1 loss compromises mitochondrial energy production in axons

In Drp1cKO mice, the synaptic deficits and disrupted mitochondrial morphology suggest that mitochondria lacking Drp1 may have impaired bioenergetic function. To examine the effects of Drp1 loss on energy levels, we tested the effect of Drp1 deletion (Drp1KO) on respiration and glycolysis in immortalized MEFs with a Seahorse Extracellular Flux (XF) Analyzer (Supplementary Figure 4). By comparing Drp1KO and control lines, we found that respiration (basal and maximal, after treatment with the uncoupler FCCP) and glycolysis (basal and after treatment with the ATP synthase inhibitor oligomycin) were unaffected by Drp1 loss (Supplementary Figure S4a and S4b). These findings are consistent with prior studies in which cell lines failed to reveal deficits in the mitochondrial membrane potential or respiration of Drp1KO mitochondria,^12, 13^ and indicate that Drp1 is not required for normal respiratory function in cell lines.

**Figure 4:**
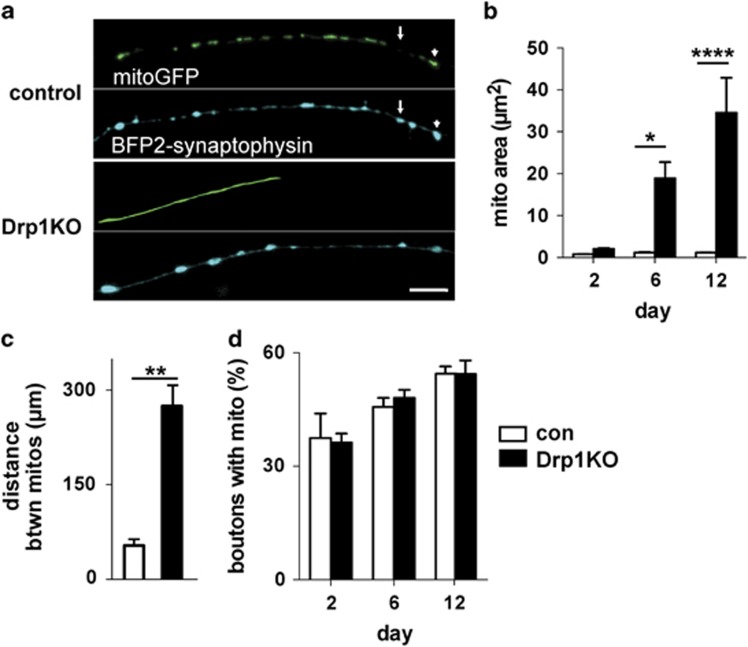
Drp1KO disrupts the distribution of mitochondria at the nerve terminal in cultured neurons. Primary hippocampal cultures from floxed Drp1 mice were co-transfected with either mCherry-Cre (to delete Drp1 (Drp1KO)) or mCherry (control), mitochondria-targeted GFP (to visualize mitochondria), and BFP2-synaptophysin (to distinguish axons from dendrites). (**a**) Drp1KO and control synapses with (arrowhead) and without (arrow) mitochondria. (**b**) Drp1KO axons had significantly bigger mitochondria. Data are means±S.E.M.; *P<0.05 and ****P<0.0001 *versus* respective controls by two-way ANOVA with repeated-measures and Sidak *post hoc* test, *n*=4–5 coverslips/group. (**c**) Drp1KO also markedly increased the distance between axonal mitochondria. **P<0.01, *versus* control by unpaired two-tailed *t*-test, *n*=3 coverslips/group. (**d**) The proportion of boutons occupied by mitochondria was unchanged. ns by two-way ANOVA, *n*=4–5 coverslips/group. Scale bar is 10 *μ*m

Neurons, however, are preferentially vulnerable to deficits in mitochondrial dynamics.^5, 6, 7, 22^ Drp1KO neurons have lower mitochondrial membrane potential at the cell body in culture,^4^ and cerebellar neurons lacking Drp1 show reduced staining of complex I and IV activity before degeneration *in vivo*.^14^ These results suggest neurons selectively require mitochondrial fission for energy production. To determine whether Drp1 is required for normal mitochondrial bioenergetic function in neurons, we examined the effects of Drp1 loss on ATP levels in postnatal hippocampal cultures from floxed Drp1 mice.^13^ We examined how Drp1 loss affects the distribution of mitochondria in axons by co-transfecting cells with either mCherry-Cre (to delete Drp1) or mCherry control, mitoGFP (to visualize mitochondria), and BFP2-synaptophysin (to distinguish axons from dendrites). Loss of Drp1 markedly increased the size of mitochondria within axons (Figures 4a and b) and the distance between mitochondria (Figure 4c). These findings are similar to those in studies that knocked down Drp1 with RNAi.^23^ The percentage of synaptic boutons containing mitochondria was unchanged (Figure 4d), consistent with our *in vivo* results (Figure 3l).

**Figure 5:**
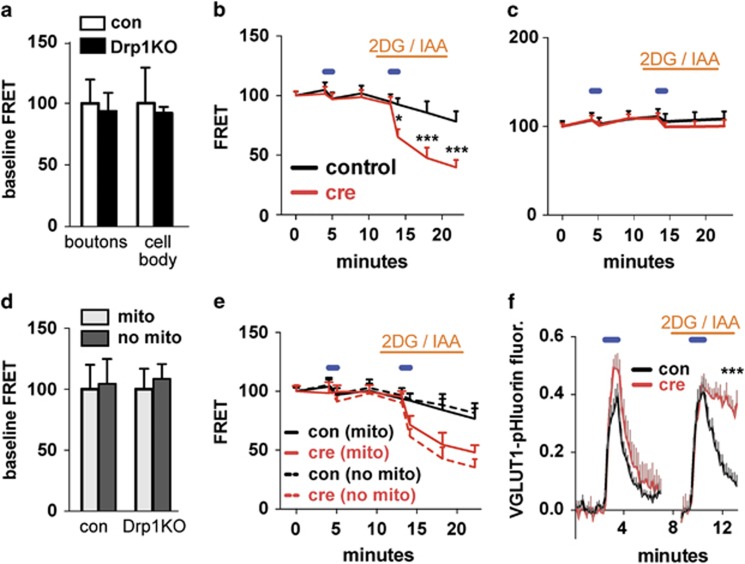
Drp1 is required for normal mitochondrial bioenergetics, specifically in axons. Drp1-floxed primary hippocampal cultures were co-transfected with an ATP-based FRET sensor (ATP1.03^YEMK^),^24^ mitoFarRed (to visualize mitochondria), and either a control vector (con) or Cre (Drp1KO). (**a**) At baseline, Drp1KO and control neurons in pyruvate and glucose showed similar levels of ATP in boutons and at the cell body, as measured by FRET. (**b**) In the acute absence of glucose, Drp1KO neurons showed significantly decreased ATP levels at the synapse after stimulation at 10 Hz*60 s (blue horizontal bars) when forced to fully rely on mitochondria for ATP (by blocking glycolysis with glycolytic inhibitors 2-deoxyglucose (2-DG) and iodoacetate (IAA) (orange horizontal bar)). (**c**) ATP levels were not depleted at the cell body, even when glycolysis was inhibited. (**d** and **e**) ATP levels were not changed in synaptic boutons, with and without mitochondria, at baseline or after stimulation. Data are means±S.E.M.; *P<0.05, ***P<0.001 *versus* control by two-way ANOVA with repeated-measures and Sidak *post hoc* test, *n*=10 coverslips/group, (with 80–140 boutons and 10–15 cell bodies sampled per group)). (**f**) VGLUT1-pHluorin was used to measure synaptic vesicle cycling in individual synapses. Drp1KO markedly impaired endocytosis when glycolysis was blocked. Data are means±S.E.M.; ***P<0.001 *versus* control group by unpaired two-tailed *t*-test for extent of endocytosis ((amplitude endocytosis)/(amplitude exocytosis)). *n*=8–10 coverslips/group, (86–111 boutons per group)

To assess how loss of mitochondrial fission affects intrinsic bioenergetic function in neurons, we co-transfected Drp1^lox/lox^ hippocampal neurons with Cre recombinase (to delete Drp1) or a vector control, the ATP-based FRET sensor (ATP1.03^YEMK^),^24^ and mitoFarRed (to visualize mitochondria). We examined ATP levels when neurons were in Tyrodes buffer with standard high glucose (~30 mM, greater than extracellular brain glucose levels, which are ~1–1.5 mM ^25, 26^) and 10 mM pyruvate (where glycolysis or respiration supports ATP production^27^). As expected, Drp1KO failed to change baseline ATP levels at the cell body or synapse (Figure 5a).

Next, we determined whether Drp1KO mitochondria have normal bioenergetic function. We examined ATP levels in the acute absence of glucose and with glycolytic inhibitors (2.5–5 mM 2-deoxyoglucose, 1 mM iodoacetate) to force neurons to rely on mitochondrial ATP. Under these conditions, Drp1KO axons could not maintain their ATP levels after acute electrical stimulation (10 Hz for 60 s), which augments ATP requirements in axons by increasing synaptic vesicle cycling,^27^ (Figure 5b). Therefore, Drp1KO mitochondria cannot maintain ATP levels in axons when energy requirements are increased. In contrast, Drp1KO mitochondria maintained ATP levels at the cell body (Figure 5c), suggesting axonal mitochondria have less capacity to maintain ATP levels than those at the cell body and/or that neural activity selectively increases ATP consumption in axons.

Why does Drp1KO decrease mitochondria-derived ATP in axons? Perhaps Drp1KO mitochondria have intrinsic deficits in their ability to produce ATP. Alternatively, axonal mitochondria are farther apart in Drp1KO neurons (Figure 4c), which may create regional gradients of ATP between them. To distinguish these possibilities, we examined ATP levels in synapses, with and without mitochondria (Figures 5d and e). Surprisingly, despite increased separation between mitochondria in Drp1KO axons, ATP levels were similar in boutons with and without mitochondria. These results suggest that Drp1KO axonal mitochondria have intrinsic deficits in their ability to produce ATP.

To determine whether decreased ATP in Drp1KO synapses is significant, we examined its impact on synaptic vesicle cycling, a key function that consumes much ATP in axons.^27^ To observe synaptic vesicle cycling in individual boutons, we used a VGLUT1-pHluorin reporter with a pH-sensitive GFP targeted to the lumen of synaptic vesicles.^28^ In the acidified lumen of the vesicle, pHluorin does not fluoresce; however, after fusing with the plasma membrane at the synapse and becoming exposed to the alkaline environment, pHluorin fluoresces and is re-quenched with re-internalization and re-acidification of the vesicle.^28^ Without glycolysis, mitochondrial-derived ATP is required to support pre-synaptic ATP levels and maintain endocytosis.^27^ However, deleting Drp1, while simultaneously inhibiting glycolysis, completely blocked endocytosis after 10 Hz × 60 s stimulation (Figure 5f), which promotes the preferential release of vesicles in the recycling pool.^29^ Therefore, mitochondria require Drp1 for normal ATP production in axons, and its loss decreases mitochondria-derived ATP sufficiently to impair synaptic vesicle cycling. These bioenergetic defects may underlie the electrophysiologic and functional deficits in Drp1cKO mice *in vivo*.

## Discussion

Disrupted mitochondrial dynamics may contribute to the pathophysiology of neurologic disorders that affect several distinct neuronal populations.^1, 9, 10, 11^ However, little is known about why changes in the fission–fusion balance affect neurons more than other cells, or why some neuronal populations are more susceptible than others. Here, we showed that CA1 hippocampal neurons are more resistant to Drp1 loss than previously studied neuronal types, although they still require Drp1 for normal synaptic function. We showed that Drp1 loss in hippocampal neurons compromises the intrinsic function of axonal mitochondria, causing significant deficits in their ability to maintain normal ATP levels and synaptic vesicle cycling.

### Mitochondrial fission is required for the bioenergetic function of axonal mitochondria

Why are neurons vulnerable to disruptions in mitochondrial fusion and fission proteins?^5, 6, 7, 22^ Mitochondrial fission facilitates targeting of mitochondria down long distal processes, and loss of this targeting likely contributes to neuronal vulnerability.^3, 4^ However, impaired axonal targeting cannot explain deficits in CA1 hippocampal neurons; these cells maintain mitochondria in axons, even without Drp1. Instead, we showed that Drp1 is required for intrinsic respiration of mitochondria in axons, as Drp1KO mitochondria cannot maintain normal ATP levels in their axons when under increased-energy demands.

Several reasons may explain why mitochondrial fission is required specifically for axonal bioenergetics. First, disrupted fission may compromise the intrinsic function of axonal mitochondria, possibly by changing their normal turnover,^30^ which could cause dysfunctional mitochondria to accumulate. Indeed, the rate of mitochondrial turnover may differ greatly between mitochondria in axons and the cell body.^31^ Second, axonal bioenergetics may be particularly sensitive to changes in mitochondrial distribution. Drp1KO markedly increased the space between axonal mitochondria in culture, which might cause regional energy gradients that would not occur at the cell body. However, we found that mitochondria-derived ATP levels were similar in Drp1KO boutons with and without mitochondria, despite the increased distance between mitochondria. Whether the distance between mitochondria may be even greater *in vivo* to potentially create these gradients is unknown. Nonetheless, these data suggest that changes in the distribution of axonal mitochondria alone cannot explain the susceptibility of axons to Drp1 loss.

Third, Drp1KO compromises the intrinsic function of mitochondria in the cell body and axons, but the energy demands are greater in axons. Indeed, synaptic transmission is the primary consumer of energy in the brain,^32^ and in particular, synaptic vesicle cycling appears to require large amounts of energy.^27^ The disproportionate energy needs in axons may contribute to their early loss in neurodegenerative diseases involving energy failure and in mitochondrial models of these diseases.^4, 33, 34, 35, 36^ In addition, the energy requirement in axons may vary considerably between neuronal types. For instance, SN DA neurons have particularly long projections, large axonal arbors, and poor myelination,^37, 38^ which might require more energy than other neuronal populations.

### CA1 hippocampal neurons are relatively resistant to Drp1 loss

Better understanding why different neuronal populations have unique requirements for specific mitochondrial fission and fusion proteins could shed light on the normal functions of mitochondrial dynamics in the brain and on how disruptions of these functions contribute to neurodegeneration.^1^ For example, cerebellar Purkinje cells die within 6 months of losing Drp1,^14^ but most midbrain DA neurons die within 1 month.^4^ However, a subset of midbrain DA neurons and their axons are far more resistant to Drp1 loss and survive with very few axonal mitochondria.^4^ Here, we showed that CA1 hippocampal neurons are also resistant to Drp1 loss, with most surviving to at least 1 year. However, in contrast to resistant midbrain DA neurons,^4^ Drp1 loss fails to decrease the number of mitochondria in CA1 axon terminals. Although some CA1 terminals might have actually lost mitochondria but atrophied before examination at 7 months, mitochondria within the surviving CA1 terminals sharply contrast with our observations in Drp1KO midbrain dopaminergic terminals, where surviving axons lose almost all of their mitochondria.^4^

Why Drp1 loss differentially affects axonal mitochondria in these two neuron types is unknown. Hippocampal neurons might have distinct pathways for mitochondrial fission that are not present in midbrain DA neurons. However, this seems unlikely: deleting Drp1 impacts mitochondrial morphology at the cell body similarly in both cell types. Alternatively, mitochondria may be preferentially lost from midbrain DA axons because they are longer and more susceptible to differences in axonal motility. However, axonal length alone cannot explain the differences, as even the proximal axons of nigrostriatal DA neurons lacking Drp1 have fewer mitochondria.^4^ Other steric or undefined differences in mitochondria and/or axons may contribute to differences in axonal targeting. We believe mitochondria in CA1 axons reflect the continued ability of mitochondria to reach the axon after Drp1 is lost, rather than an inability to degrade those mitochondria that reached the nerve terminal before Drp1 was lost. Indeed, we examined the synaptic mitochondria >4 months after appearance of the characteristic swollen mitochondria with Drp1 loss at the cell body, a time interval that far exceeds estimates of mitochondrial lifespan.^39^ Nonetheless, we cannot exclude this possibility, as very little is known about mitochondrial lifespan in neurons and their axons, and Drp1KO could prolong their lifespan by impairing mitochondrial turnover.^40^

### Implications for Drp1 in the pathophysiology of neurologic diseases

We showed that CA1 hippocampal neurons – susceptible to degeneration in AD, stroke, and seizure disorders^41, 42, 43^ – require Drp1 for respiration and synaptic function. Interestingly, Drp1 has been mechanistically linked to the pathogenesis of AD in several studies. For example, amyloid-beta may mediate toxicity by increasing Drp1 function and producing excessive fission.^44^ In contrast, mutant tau produces toxicity by downregulating Drp1 in *Drosophila*.^45^ With the current study, these findings suggest that too much and too little fission are detrimental and that normal neuronal functions may depend on a fine balance between mitochondrial fission and fusion. Changes in mitochondrial fission may also affect the pathophysiology of stroke and seizure, in which Drp1 is a therapeutic target.^9, 10, 11^ Furthermore, AD, seizures, and stroke are associated with increased synaptic transmission and impaired metabolism,^31, 46, 47, 48^ and changes in mitochondrial fission might contribute to or synergize with these alterations.

Our Drp1cKO mouse provides an excellent model to study the effects of mitochondrial fission in these neurological disorders. This model is the first to both avoid early developmental changes and separate the normal functions of Drp1 from cell death processes, which could confound analyses. Building upon the physiological functions of Drp1 identified here, the Drp1cKO model can now be used to elucidate the role of this protein in models of neurological disorders that target the hippocampus.

## Materials and Methods

### Animals

Floxed Drp1 mice have been described.^13^ CamKCre^15^ and tdTomato mice^17^ were obtained from Jackson Laboratory. Mice were group-housed in a colony maintained with a standard 12 h light/dark cycle and given food and water *ad libitum*. Experiments were performed on age-matched mice of either sex. No differences between sexes were noted in any of the experiments. Experiments were conducted according to the *Guide for the Care and Use of Laboratory Animals*, as adopted by the National Institutes of Health, and with approval of the University of California, San Francisco, Institutional Animal Care and Use Committee.

### Behavioral testing

Learning and memory was assessed with the Morris water maze (MWM) test.^49^ In brief, mice underwent two sessions of hidden-platform training separated by a 2 h intersession rest. Each session consisted of two trials. This training was performed each day for 5 days. The platform was removed and memory probe trials were performed 24 and 72 h after the last training day. Three sessions of visible platform training were performed the next day after training and probe trials were complete, as a control.

EthoVision video-tracking system (Noldus, Wageningen, the Netherlands) was used to record and track mice. Open field locomotor activity was performed as described.^4^ In brief, mice were habituated for at least 1 h before recording activity for 15 min with an automated Flex-Field/Open Field Photobeam Activity System (San Diego Instruments, San Diego, CA, USA). All behavioral experiments were performed with the examiner blind to genotype.

### Slice preparation and electrophysiology

Transverse hippocampal slices were cut at 400 *μ*m as described.^50^ In brief, mice were anesthetized by isoflurane inhalation and killed by decapitation. Their brains were isolated and immediately placed in an ice-cold solution containing 234 mM sucrose, 2.5 mM KCl, 1.25 mM NaH_2_PO_4_, 10 mM MgSO_4_, 26 mM NaCO_3_, 11 mM glucose, and 1.3 mM ascorbic acid. Brains were sliced on a Leica VS100 vibroslicer (Leica, Nussloch, Germany). Slices were incubated for at least 1 h in oxygenated artificial cerebrospinal fluid (aCSF) containing 126 mM NaCl, 2.5 mM KCl, 1.25 mM NaH_2_PO_4_, 1 mM MgSO_4_, 26 mM NaCO_3_, 10 mM glucose, and 2 mM CaCl_2_ at room temperature (RT) before being transferred to a submerged recording chamber. Each slice was equilibrated for 10–20 min before recording. For extracellular field EPSP recordings, aCSF-filled glass pipette was placed in the stratum radiatum, and the Schaffer-collateral pathway was stimulated with a concentric bipolar electrode. Electrophysiological recordings were filtered, digitized, and acquired with WinLTP (Bristol, UK, RRID:nif-0000-31907). Analysis was performed with WinLTP and Original pro 8.0 (Origin Labs, Northampton, MA, USA).

### Stereotaxic injection of recombinant adeno-associated virus (AAV)

Intracranial injections of AAV1-EF1*α*-DIO-mitoGFP (8 × 10^12^ VG/ml) or mCherry-synaptophysin (3 × 10^12^ VG/ml)^4^ were performed in ~6-month-old CamKCre or Drp1cKO mice. Mice were anaesthetized with 2,2,2-tribromoethanol (Alfa Aesar, Ward Hill, MA, USA; 500 mg/kg) and secured with a stereotaxic frame (Kopf, Tujunga, CA, USA). Viruses were mixed at a 1 : 1 ratio and then injected unilaterally (0.5 *μ*l) into the CA1 (A/P, 2.1 mm from bregma; M/l, 2 mm; D/V from skull, 1.4 mm) at a rate of 0.2 *μ*l/min with a Hamilton syringe and cannula (33 gauge). Animals were killed at 7 months of age. Quantification was performed by ImageJ software and the 'Analyze Particles' plug-in for synaptic and mitochondrial size, and the ImageJ colocalization plug-in for mitochondrial occupancy at the synapse (RRID:nif-0000-30467).

### Histology

For histology experiments, mice were anaesthetized and perfused with phosphate-buffered saline (PBS), and then 4% paraformaldehyde. Brains were then removed, postfixed in PFA (overnight, or for 2 h for Drp1 staining), and cryoprotected in 30% sucrose. Coronal brain slices (30 or 50 *μ*m for Drp1 levels) were prepared using a sliding microtome (Leica SM2000R). To analyze hippocampal volume, brain slices (40 *μ*m thickness) were frozen in superchilled isopentane and prepared with a Leica cryostat (Leica CM1900).

For immunofluorescence, sections were blocked for ≥1 h in PBS with 0.2% Triton X-100 and 10% bovine calf serum and then incubated with primary antibodies overnight at RT. The following primary antibodies were used: mouse anti-Drp1 (1 : 200; BD Biosciences, San Jose, CA, USA; clone 8); chicken anti-MAP2 (1 : 1500; Abcam, Cambridge, MA, USA; Cat# ab5392 RRID:AB_2138153); mouse anti-NeuN (1 : 1000; Millipore, Darmstadt, Germany; Cat# MAB377 RRID:AB_2298772); rabbit anti-Tom20 (1 : 500; Santa Cruz, Dallas, TX, USA; Cat# SC-11415, RRID:AB_2207533); mouse anti-MAP2 (1 : 1000; Millipore Cat# MAB3418 RRID:AB_94856); rabbit anti-calbindin (1 : 20000; Swant, Marly, Switzerland; Cat# 300 RRID:AB_10000347); rabbit anti-dsRed (1 : 1000; Clontech, Mountain View, CA, USA; Cat# 632496 RRID:AB_10015246). Sections were rinsed and incubated for 2 h at RT with the corresponding secondary antibodies: Alexa Fluor 488, 594, or 647 anti-mouse, chicken, or rabbit IgG (1 : 250–1 : 500; Invitrogen, Grand Island, NY, USA). For peroxidase staining, sections were incubated with rabbit anti-dsRed, followed by biotinylated goat anti-rabbit IgG (1 : 300; Vector Laboratories, Burlingame, CA, USA; BA-1000, RRID:AB_2313606), and subsequently streptavidin-conjugated horseradish peroxidase (1 : 300; Vectastain ABC kit, Vector Laboratories). Immunostaining was visualized with hydrogen peroxide and 3,3′-diaminobenzidine (DAB, Sigma, St. Louis, MO, USA).

Brain sections were imaged with a laser-scanning confocal microscope (LSM510-Meta; Carl Zeiss, Jena, Germany) with a × 63 (1.4 NA) PlanApo oil objective, a Nikon Ti-E inverted microscope with a × 60 (1.2 NA) PlanApo water objective, or a Keyence inverted microscope BZ-9000 with a × 10 (0.45 NA) CFI PlanApo *λ* objective. Volume was calculated with the Cavalieri principle.^51^ Quantification of fluorescence and area was performed blind to genotype with MetaMorph software (version 7.7.3.0; Molecular Devices, Sunnyvale, CA, USA; RRID:SciRes_000136). Neuronal density was calculated by dividing the total fluorescence of NeuN in a fixed area in CA1 (a surrogate for the total number of neurons in this area) by the average NeuN intensity per CA1 neuron. Quantification of cells with swollen mitochondria was scored blind to genotype, based on the presence of three or more swollen mitochondria in a cell (a subjective criteria chosen to distinguish Drp1cKO *versus* control mitochondria).

### Neuronal culture and live imaging

Postnatal hippocampal neuronal cultures were prepared from P0 Drp1^lox/lox^ mice as described^4^ and transfected via electroporation (Amaxa; Lonza, Basel, Switzerland) with one or more of the following constructs, all expressed in the pCAGGS vector downstream of the chicken actin promoter:^28^ ATP-YEMK (kind gift of Dr Noji, Osaka University),^24^ mCherry-synaptophysin,^52^ BFP2-synaptophysin, VGLUT1-pHluorin-ires-mCherry-synaptophysin,^28, 52^ Cre recombinase,^4^ mCherry-Cre, mCherry, mitoGFP,^53^ mitoFarRed, or mitoTagBFP. mitoFarRed and mitoTagBFP were generated by fusing TagRFP657 or TagBFP (kind gifts from Vladislav Verkhusha, Albert Einstein), respectively, to the mitochondria-targeting sequence, cytochrome C oxidase subunit VIII.^54, 55^ Neurons were cultured for 8–11 days before live imaging or analysis.

Live imaging was performed in Tyrode's medium (pH 7.4; 127 mM NaCl, 10 mM HEPES-NaOH, 2.5 mM KCl, 2 mM MgCl_2_, and 2 mM CaCl_2_ with 30 mM glucose and/or 10 mM pyruvate) on a Nikon Ti-E inverted microscope with an iXon EMCCD camera (Andor Technology, Belfast, UK) and a perfusion valve control system (VC-8, Warner Instruments, Hamden, CT, USA) controlled by MetaMorph Software. Field stimulation (10 Hz × 60 s) was performed with an A385 current isolator and a SYS-A310 accupulser signal generator (World Precision Instruments, Sarasota, FL, USA). Glycolysis was inhibited with 2-DG (2.5–5 mM, Sigma-Aldrich, St. Louis, MO, USA) and iodoacetate (1 mM, Sigma-Aldrich).

VGLUT1-pHluorin fluorescence images were obtained (490/20 ex, 535/50 em, Chroma, Bellows Falls, VT, USA) and regions of interest were drawn over synaptic boutons with MetaMorph software. Synaptic boutons were identified based on colocalization with mCherry-synaptophysin and an increase in pHluorin fluorescence after applying ammonium chloride (50 mM). For each bouton, the background-subtracted change in fluorescence at each time point was normalized to the fluorescence in ammonium chloride (which estimates the total size of the synaptic vesicle pool)^29^ measured at the end of each run. The baseline fluorescence intensity was set to zero at *t*=100 s before the first stimulation and *t*=170 s before the second stimulation.

For FRET experiments, sequential images were taken in the CFP (430/24 ex, 470/24 em), YFP (500/20 ex, 535/30 em), and FRET channels (430/24 ex, 535/30 em) with an ET ECFP/EYFP filter set (Chroma). Synaptic boutons were identified based on colocalization with mCherry-synaptophysin or determined by morphology for experiments with mitoFarRed. Synaptic boutons were classified as containing or lacking mitochondria based on images taken immediately before and after the imaging run. The FRET/donor ratio was calculated for each bouton as described,^56^ where FRET=(I_FRET_ − I_CFP_ × BT_CFP_—I_YFP_ × BT_YFP_)/I_CFP_, such that I_X_ is the background-corrected fluorescence intensity measured in a given channel. BT_CFP_ (donor bleed through) and BT_YFP_ (direct excitation of the acceptor) were calculated by expressing CFP and YFP individually and determining the ratios of I_FRET_/I_CFP_ and I_FRET_/I_YFP_, respectively.

### Respiration and glycolysis in MEFs

Respiratory and glycolytic rates in four wild-type and four knockout MEF cell lines were measured with the XF Analyzer 96-well plate reader. MEFs were seeded at 20 000 cells/well 24 h before reading. Then, they were incubated in Seahorse DMEM with 30 mM glucose 1 h before and also during metabolic readings. Respiration and glycolysis were simultaneously measured based on oxygen consumption rates and media acidification, respectively. Oligomycin (1*μ*M) was injected, followed by FCCP (1 *μ*M) and then rotenone (1 *μ*M). Metabolic rates were normalized to cell numbers based on DAPI counts.

### Statistical analysis

To analyze learning by MWM testing, we fit a linear mixed effects model^20^ of the natural log of the distance traveled on time (trial) using the R Project for Statistical Computing (RRID:nif-0000-10474) package lme4. To allow for nonlinearity in the curves, we included a quadratic effect of time. We included fixed intercepts and slopes for each of the two genotypes, each of the two age groups, possible interactions of these effects, and random intercepts for each mouse to account for the correlation among repeated observations.

The assumptions of a normal distribution (normality) and equal variance among the groups (homoscedasticity) were checked by looking at the distribution of the residuals (difference between observed and fitted values) and the fitted values from the model. A natural logarithmic transformation of the response variable was deemed necessary as the assumptions of normality and homoscedasticity were violated. The estimates from the model were back transformed to the original scale.^57^

We used the fitted model to obtain estimates of the mean distance on the fourth and eighth trials for each group. We used the function sim() from the arm package to obtain 5000 simulations of the estimates and compute a 95% confidence interval (CI) around each estimate as the 2.5th and 97.5th quantiles of these draws. We calculated *P*-values for differences between groups by inverting the simulated CIs around the differences.^58^ These *P*-values were corrected for multiple comparisons using the method of Holm.

## Abbreviations

Drp1: dynamin-related protein 1
AD: Alzheimer's disease
Cre: CamKIIalpha-Cre
Drp1cKO: Cre recombinase Drp1^lox/lox^
Drp1KO: Drp1 knockout
Opa1: optic atrophy 1
Mfn2: mitofusin-2
GDAP1: ganglioside-induced differentiation-associated protein 1
PD: Parkinson's disease
MEFs: mouse embryonic fibroblasts
CamKCre: CamKIIalpha-Cre
EPSPs: excitatory post-synaptic potentials
EC: entorhinal cortex
MWM: Morris water maze
aCSF: artificial cerebrospinal fluid
RT: room temperature
HRP: horseradish peroxidase
DAB: 3,3′-diaminobenzidine
XF: Seahorse Extracellular Flux
CI: confidence interval
2-DG: 2-deoxyglucose
IAA: Iodoacetate
Ns: not significant
Oligo: oligomycin
Rot: rotenone
WT: wild-type
KO: knockout
DG: dentate gyrus
AAV: adeno-associated virus
FCCP: carbonyl cyanide-4-(trifluoromethoxy)phenylhydrazone
DA: dopaminergic
