# Supplementary material for: Dynamin-related protein 1 is required for normal mitochondrial bioenergetic and synaptic function in CA1 hippocampal neurons
Source: Cell Death Dis. 2015 Apr 16;6(4):e1725–. doi: 10.1038/cddis.2015.94 (PMC4650558; doi:10.1038/cddis.2015.94)
Supplement: Supplementary Figures [file cddis201594x1.pdf]

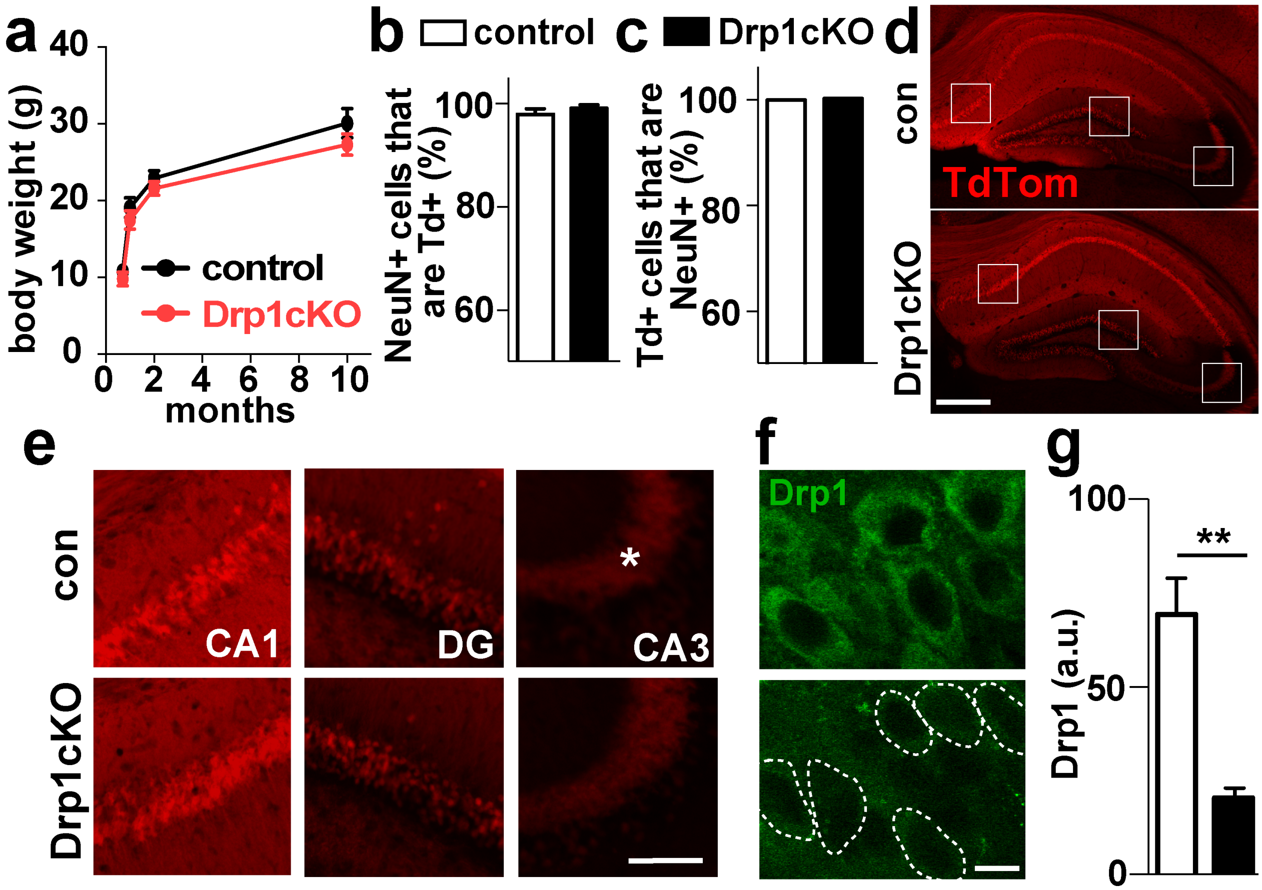

Supplementary Figure 1. Cre-expression and Drp1 loss in the hippocampus. (**a**) Weights of Drp1cKO (Drp1$^{lox/lox}$;CamKII-Cre) and control (Drp1$^{lox/lox\ or\ lox/wt}$) mice were similar through 10 months. Data are means ± SEM; n= 6–14 mice/group. (**b**) Cre-expression in CA1 was assessed using tdTomato-CamKCre control (tdTomato$^{lox/wt}$;CamKII-Cre) and Drp1cKO-tdTomato-CamKCre (Drp1$^{lox/lox}$;tdTomato$^{lox/wt}$;CamKII-Cre) mice. Almost all CA1 neurons (marked by NeuN) expressed TdTomato, indicating that they expressed Cre. (**c**) All TdTomato-positive cells expressed NeuN, indicating that all cells expressing Cre are neurons. Data are means ± SEM; n=3 mice/genotype, 4–6 slices/mouse. (**d**) TdTomato was expressed by neurons in CA1 and the dentate gyrus (DG) (≈ 50%), with very little expression in CA3. Scale bar is 400 μm. (**e**) Enlarged insets of CA1, CA3 and DG, indicated by white boxes in (**d**). Almost no TdTomato expression was detected in CA3 neurons, but fibers synapsing on CA3 can be visualized and are marked by an asterisk. Scale bar is 100 μm. (**f**) Drp1 staining of brain sections from 1-year-old control and Drp1cKO mice. Outlines of cell bodies were defined by co-staining with MAP2 (not shown). Scale bar is 10 μm. (**g**) CA1 neurons from Drp1cKO mice had markedly lower Drp1 levels (quantified per cell body) than Drp1WT (control). Data are means ± SEM; **p<0.01 by unpaired two-tailed *t* test, n=4 mice/genotype, 4–6 slices/mouse.

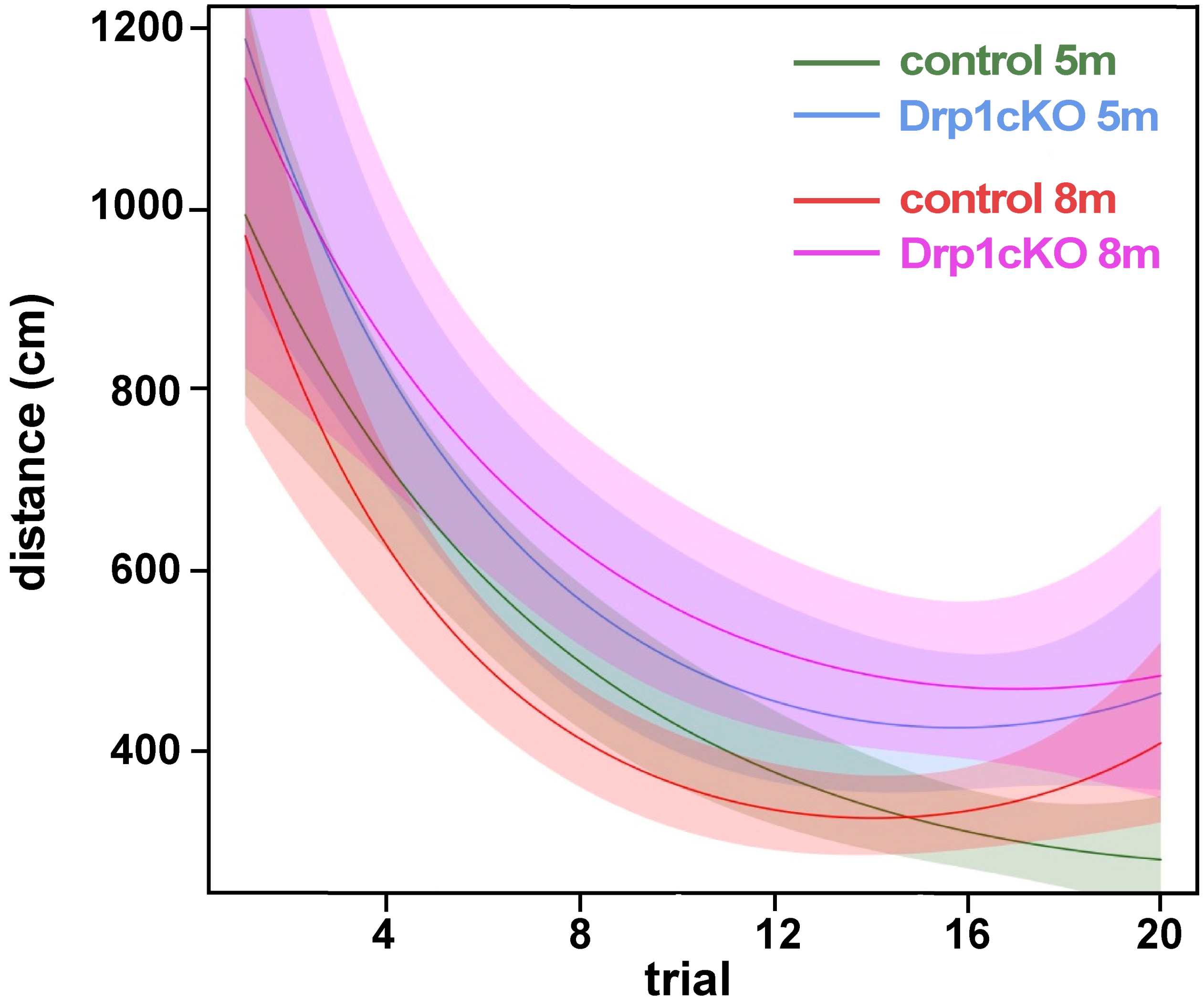

Supplementary Figure 2. Linear mixed effect modeling of Drp1cKO and control learning throughout Morris water maze. Based on the linear mixed effects model, we estimated hidden distance trajectories for each genotype (i.e., Drp1WT (control) and Drp1cKO) at 5 and 8 months over trials 1–20; 95% confidence intervals are indicated by the shading around each curve.

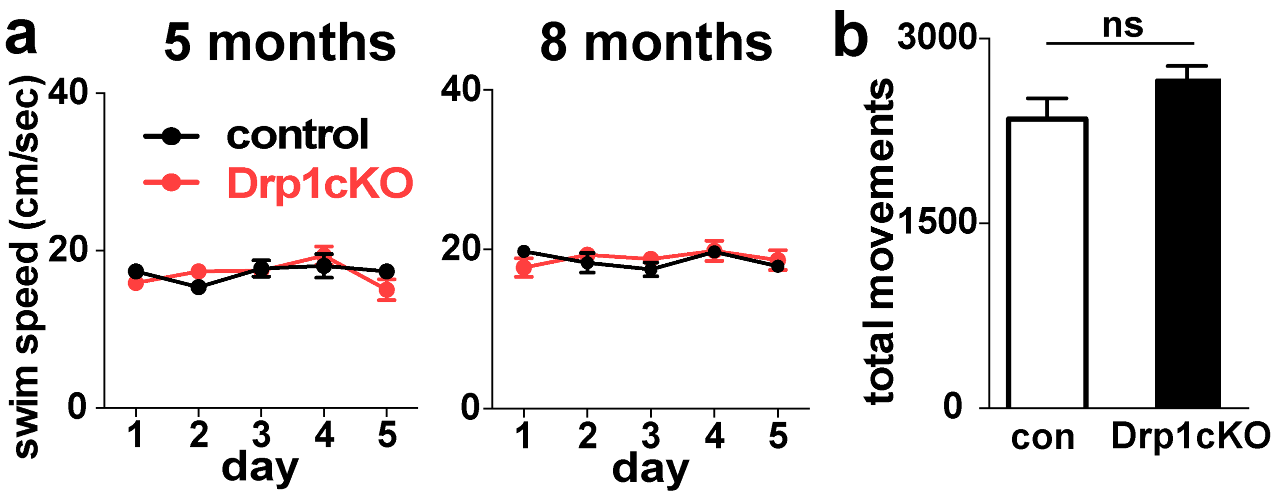

Supplementary Figure 3. Drp1 loss does not affect motor activity in vivo. **a**) Drp1cKO and Drp1WT (control) mice showed no differences in swim speed during 5 days of hidden Morris water maze training, suggesting that differences in test results were not due to motor deficits. (**b**) Motor activity in open field showed no difference between the groups. Total movements of 4–month-old control and Drp1cKO mice were measured for 15 min in open field. Data are means ± SEM; ns=not significant by unpaired two-tailed *t* test, n=7 mice/group.

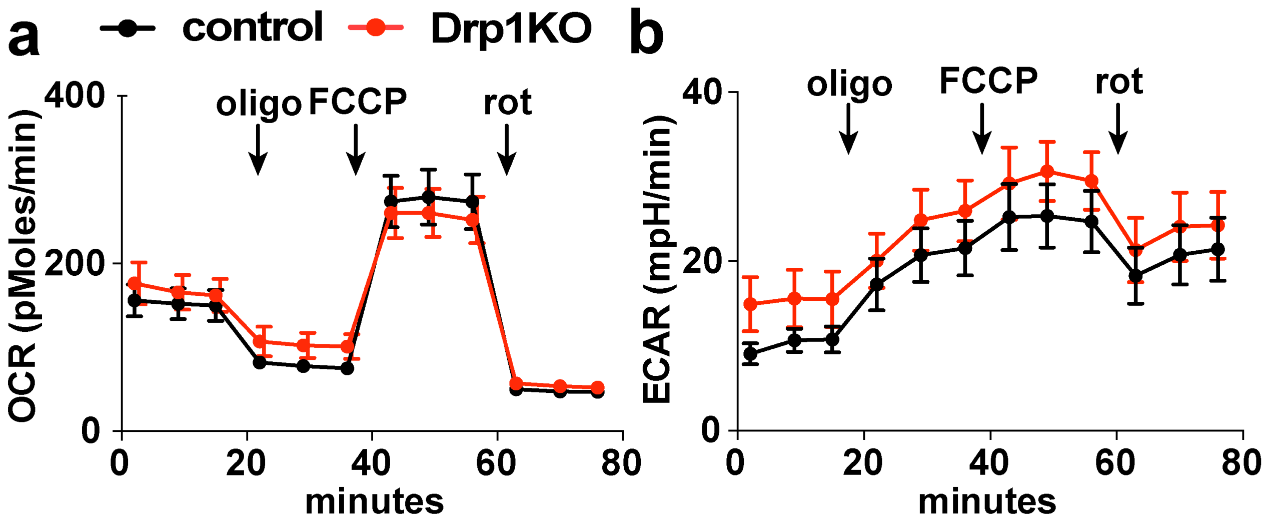

Supplementary Figure 4. Drp1 loss does not alter respiration or glycolysis in mouse embryonic fibroblasts (MEFs). MEFs lacking Drp1 were cultured, and mitochondrial respiration and glycolysis were measured with a 96-well Seahorse Extracellular Flux Analyzer. (**a**) Drp1KO did not affect basal or maximal (post-FCCP) respiration rates. (**b**) Drp1KO did not significantly affect the rate of extracellular acidification, a surrogate for glycolysis. Oligomycin (oligo, 1 μM) blocks ATP synthase, FCCP (1 μM) uncouples mitochondria, and rotenone (rot, 1 μM) blocks complex I. Data show the average metabolic rates of four wild-type (WT) and knock-out (KO) MEF cell lines, normalized to cell number determined by DAPI staining. Data are means ± SEM; n=8 cell lines/group (18 wells/cell line).
